# Selection of data sets for FAIRification in drug discovery and development: Which, why, and how?

**DOI:** 10.1016/j.drudis.2022.05.010

**Published:** 2022-08

**Authors:** Ebtisam Alharbi, Yojana Gadiya, David Henderson, Andrea Zaliani, Alejandra Delfin-Rossaro, Anne Cambon-Thomsen, Manfred Kohler, Gesa Witt, Danielle Welter, Nick Juty, Caroline Jay, Ola Engkvist, Carole Goble, Dorothy S. Reilly, Venkata Satagopam, Vassilios Ioannidis, Wei Gu, Philip Gribbon

**Affiliations:** a Department of Computer Science, The University of Manchester, Oxford Road, Manchester, UK; b College of Computer and Information Systems, Umm Al-Qura University, Mecca, Saudi Arabia; c Fraunhofer Institute for Translational Medicine and Pharmacology (ITMP), Schnackenburgallee 114, 22525 Hamburg, and Theodor Stern Kai 7, 60590 Frankfurt, Germany; d Fraunhofer Cluster of Excellence for Immune Mediated Diseases (CIMD), Theodor Stern Kai 7, 60590 Frankfurt, Germany; e Bayer AG, Research & Development, Pharmaceuticals, Müllerstrasse 178, 13353 Berlin, Germany; f CERPOP, UMR 1295, INSERM, Université Paul Sabatier -Toulouse III, France; g Luxembourg Centre for Systems Biomedicine, ELIXIR Luxembourg, University of Luxembourg, L-4367 Belval, Luxembourg; h Discovery Sciences, R&D, AstraZeneca, SE-43183 Mölndal, Sweden; i Novartis Institutes for BioMedical Research, Novartis Pharma AG, Basel, Switzerland; j SIB Swiss Institute of Bioinformatics, Quartier Sorge - Batiment Amphipole, 1015 Lausanne, Switzerland

**Keywords:** FAIR, FAIRification, Pharmaceutical R&D, Drug discovery, Cost–benefit, FAIR, Findable, Accessible, Interoperable, and Reusable, GDPR, General Data Protection Regulation, R&D, Research and Development, DPIA, Data Protection Impact Assessment, RDM, Research Data Management, DMP, Data Management Plan, EFPIA, European Federation of Pharmaceutical Industries and Associations, DTA, Data Transfer Agreement

## Abstract

Despite the intuitive value of adopting the Findable, Accessible, Interoperable, and Reusable (FAIR) principles in both academic and industrial sectors, challenges exist in resourcing, balancing long- versus short-term priorities, and achieving technical implementation. This situation is exacerbated by the unclear mechanisms by which costs and benefits can be assessed when decisions on FAIR are made. Scientific and research and development (R&D) leadership need reliable evidence of the potential benefits and information on effective implementation mechanisms and remediating strategies. In this article, we describe procedures for cost–benefit evaluation, and identify best-practice approaches to support the decision-making process involved in FAIR implementation.

## Highlights

• Research organisations are focussed on quantifying the costs and benefits of implementing FAIR.
• Criteria used for the selection of data for FAIRification can be opaque and inconsistent.
• FAIRification effort depends on individual skills, competencies, resources, and time available.
• FAIRification should satisfy reuse scenarios, and lead to scientific and economic impacts.
• Organisational challenges include providing training to individuals and developing a FAIR organisation culture.

## Introduction

The FAIR data-guiding principles provide a framework for the management of scientific research data,^1^ and offer a systematic approach to tackling the challenges of reusing fast-growing, but frequently inaccessible and inconsistently annotated, research data resources. Since their formulation in 2016, these aspirational principles have attracted considerable interest from biomedical researchers in academia and industry, who aim to foster their implementation to help improve the overall efficiency of the research process.^2^ Furthermore, major funding agencies now expect commitments toward the creation of open and reusable data resources from organisations receiving financial support from public bodies.3., 4. Implementing these principles has the potential to maximise the value of scientific data by enabling advanced analyses, such as machine learning (ML) and artificial intelligence (AI) techniques.5., 6. Recent studies emphasise that the availability of virus, patient, and therapeutic discovery data in a FAIR format could have accelerated the response to the Coronavirus 2019 (COVID-19) pandemic by enabling large-scale analysis.7., 8. Hence, FAIRifiying data and metadata is a prerequisite for attempts to elevate the overall reproducibility of research findings.^9^.

To realise the potential of FAIR research data management and to drive FAIR data adoption, several prominent collaborations between academia and industry in the life science discipline have been established. An example of FAIR implementation in this setting is the Pistoia Alliance (https://www.pistoiaalliance.org), a precompetitive pharmaceutical industry collaboration that funds activities fostering FAIR adoption.^10^ A second initiative is the FAIRplus project (https://fairplus-project.eu), an ongoing EU project developing practical guidelines and tools to FAIRify clinical and translational biomedical data. FAIRplus develops FAIRification tools as part of collaborations with Innovative Medicine Initiative (IMI) projects seeking to FAIRify their data resources, with the aim that the tools can be reused by the wider community, therefore increasing their overall impact.^11^ Both initiatives make a significant contribution to transforming data management and stewardship and drive FAIR implementation in the biomedical field.

Recent studies have described FAIR implementation attempts in the pharmaceutical industry, which primarily focussed on improving the effectiveness of the drug R&D process12., 13. and accelerating innovation within organisations.^14^ Many pharmaceutical organisations are also understandably focussed on the associated costs and expected benefits of implementing these principles, in particular for retrospective processing of legacy data, where the immediate impact is arguably less clear than for ongoing projects.^15^ Despite the recognised value of these principles, putting them into practice (the so-called ‘FAIRification process’^16^) presents significant challenges.

## FAIRification challenges

Several hurdles might hinder the effective implementation of FAIR data principles. The most-frequently cited barriers are the financial, technical, legal, and organisational aspects of implementation.13., 14., 15., 17., 18., 19., 20., 21. Financial challenges are related to the costs of the resources required to implement the FAIRification process, beginning with establishing and maintaining physical data infrastructures. They also include the significant costs of employing personnel and providing for the long-term sustainability of the data resources.15., 18. By contrast, technical challenges are associated with the infrastructure, tools, and methodologies that are required to perform FAIRification (with the help of persistent identifier services, metadata registries, ontology services, etc.).^19^ Legal challenges correspond to requirements that might pertain to the processing and sharing of the data (e.g., accessibility rights and compliance with data protection regulations), both for meeting the ‘accessibility’ and ‘reusability’ criteria as well as for performing the FAIRification process itself.20., 21. Organisational challenges include providing training to the individuals who would implement and maintain the FAIRification processes. Furthermore, these also involve the development and sustaining of an organisational culture that elevates and rewards the practice of FAIR research data management (https://rdmkit.elixir-europe.org/).^13^ All the above-mentioned challenges must be systematically addressed to implement FAIR effectively and apply equally to both retrospective and prospective processing of data sets. We define ‘data set’ as a collection of related records, of the same type, presented in a structured way. This typically includes individual files, sets of web pages, or spreadsheets. Table 1 lists the challenges that affect FAIRification along with their required expertise.

**Table 1:** FAIRification challenges and their required expertise.

**Challenges of the FAIRification process**		**Required expertise**
Financial investment	Establishing and maintaining the physical data structure Curation costs Ensure business continuity Developing a long-term plan of the data strategy	Business lead, strategy lead, associate director
Technical infrastructure	Availability of technical tools (persistent identifier services, metadata registry, ontology services, etc.)	IT professionals, data stewards, domain experts
Legal compliance	Accessibility rights Data protection regulations	Data protection officers, lawyers, legal consultants
Organisational culture	Organisational business goals Internal data management policies and plans Education and training of personnel	Data experts, data champions, data owners, IT professionals

Although some criteria used for the selection of data undergoing FAIRification can be opaque and inconsistent, the legal and ethical aspects of decision-making must take into account protection of participants’ data, respecting rights and freedoms as well as ensuring the interests of data owners. For example, Boeckhout *et al.* stated that ethical and legal aspects significantly affect the implementation of FAIR practices for sensitive human data.^17^ Similarly, Holub *et al.* identified guidelines to help compliance with legal requirements for FAIR data in health and medical research.^21^ At the outset, if personal data are involved, a thorough assessment of the access and reuse conditions of the data should be made and the requirements for compliance with General Data Protection Regulation (GDPR) and/or other applicable data protection legislation should be satisfied to ensure that the FAIRness goals do not contradict data protection principles.22., 23. A general data protection procedure, based upon the requirements of the GDPR framework,^24^ should be formulated covering aspects such as data usage, storage, and the intended purpose of analysis when personal data are involved. If the data set to be FAIRified contains sensitive personal data, such as health or racial or ethnic information, for which data protection regulations specify a stricter framework for processing,^25^ then an assessment of the suitability for FAIRification should identify the security and confidentiality requirements that must be fulfilled as part of the FAIRification process.^21^.

A Data Protection Impact Assessment (DPIA) should also be conducted to evaluate the risks of data processing and define the measures to take to address those risks and demonstrate compliance with data protection regulation.^26^ In situations in which anonymisation of data is not possible, participants’ consent should be sought and security measures (e.g., authentication procedure, rules for access, tracking of access, data encryption, etc.) should be considered to protect the privacy of individuals. FAIR Research Data Management (FAIR RDM) procedures are aligned with the necessary compliances needed when working with sensitive data, for example, access in FAIR is meant to apply only for appropriate individuals. However, it can be burdensome in some cases and recent calls have been made to refine GDPR regulations to better account for emerging reuse scenarios for sensitive data sets.^27^.

The status of a data set, including the quality and completeness of metadata, is a crucial factor influencing decision-making on implementing FAIRification, because this defines the intrinsic suitability for reuse. However, in many cases, it is not possible to predict the full range of reuse scenarios for a specific data set while designing the FAIRification objectives.^28^ It might also be the case that the scale of data involved could still be insufficient or statistically underpowered to answer meaningful scientific questions.^29^ Therefore, there may be a lower threshold of the size or scope of a FAIR data set, depending on its reusability needs, such as rare diseases research. For example, the requirements to achieve statistical power in an analysis do not change just because the underlying data set has been FAIRified. Similarly, upper thresholds might exist because of the resources needed to perform FAIRification, especially if the FAIRification procedure is not scalable or readily automatable.^19^.

The tractability of any planned data FAIRification effort depends on the skills, competencies, resources, and time available to address the specific needs of the data resource or workflow. Therefore, the availability of in-house technical data experts or champions is a crucial factor. Data champions are individuals who can provide practical insight into the selection and prioritisation decisions. To minimise the risk of misinterpretation of the data, it is also desirable to have scientific experts with domain-specific knowledge within FAIRification teams.^28^ These individuals act as a human reference, able to answer questions to provide salient context-relevant information on the data sets and their underlying properties.^30^ Domain experts collaborating with IT professionals (to provide platforms, tools, and skills to work on data), bioinformaticians, or data curators can help assess the likely impact of a planned FAIRification process in terms of the scientific or organisational advancements enabled by data reuse. Furthermore, it is crucial to clearly define the underlying goal of the FAIRification effort, especially when it relates to ‘nontechnical’ factors, such as meeting contractual obligations to funders or complying with the data management policy of an organisation.^31^ Recently, extensive guidelines have been established on the implementation of FAIR-based Data Management Plans (DMP) by the European Commission as well as national research funding organisations.^32^

The benefits that pharmaceutical organisations stand to gain from data-resource FAIRification are linked to the basic business imperative to bring safe and effective products to patients in a timely and cost-efficient manner. For this group, when considering which resources to FAIRify, the greatest potential impact will come from data associated with the complex and expensive clinical trial processes.33., 34. To convince management to invest in relatively complex FAIR working, especially with sensitive patient data, more examples are needed that show clearly how using community-derived FAIR data has an impact on real-world problems, such as selecting the best treatment regimen or trial design for a specific patient cohort.13., 35. In both drug discovery and development, FAIR data management procedures will potentiate the adoption of improved analysis methods, including ML and AI-based tools and workflows.36., 37. Thus, users will be able to generate, test, and validate general prediction models and/or processes in their specific data domain.^6^ The higher aggregation levels achievable in this way will pave the way to more-precise models of human health and disease at the molecular, cellular, tissue, and organismal levels.

The value and potential of the reuse of a FAIRified data set differ according to the perspective of the individual scientist or organisation. Personal factors are important for individual scientists who naturally seek to elevate the findability of their own research results, thereby increasing their citation rates, bringing benefits in terms of improved scientific recognition and career progression.^14^ For project teams, FAIR data management might allow for a study to progress more efficiently because fewer experiments might be necessary when FAIR community-sourced data can be substituted for planned experiments, thereby reducing unnecessary repetition of studies.15., 21., 28. The expected cumulative impact of access to FAIRified data is to achieve a higher degree of process reproducibility because of better metadata descriptions, which can reveal the source of inconsistencies between studies.

It is crucial for the success of FAIR implementation to evaluate the above-mentioned challenges, along with the value generated from FAIRification based on the principles of the cost–benefit analysis.^38^ Such an analysis is a powerful way to support decision-making by evaluating the associated costs and expected benefits in a complex situation.39., 40. A recent study introduced the FAIRification costs and the FAIRification benefits in the pharmaceutical R&D setting.^15^ It identified a set of factors that affect the costs and values of the FAIRification process to make an effective decision on data prioritisation. The cost factors refer to the set of indicators or aspects that influence the costs associated with the FAIRification process. These factors are: (i) the legal and ethical considerations, which include the access rights and the ethical compliance; (ii) the technical resources, which pertain to the availability of the IT applications needed to perform the FAIRification and the availability of the documentation, such as DMP; and (iii) the human resources, which mean having skilled personnel to carry out the FAIRification. By contrast, value factors can be defined as the value proposition for performing the FAIRification. These factors are: (i) the societal value, which concerns addressing an area of priority need or cross-cutting impact; and (ii) the scientific value, which focusses on the uniqueness and novelty of the data set, its potential synergies, covering of a domain, and the domain expert availability. Table 2 summarises the identified cost and value factors that might influence decision-making on the FAIRification process.

**Table 2:** Factors related to the cost and value of the FAIRification process.

**FAIRification cost factors**		**FAIRification value factors**	
Legal and ethical considerations	Existing or required data access/data-processing agreements Compliance with data protection regulations	Societal value	Project focus on area of priority Cross-cutting impact
Technical resources	Availability of (software) tools Quality and completeness of metadata Availability of DMP	Scientific value	Uniqueness and novelty Scientific champion Potential synergies Coverage of a domain
Human resources	Availability of internal expertise Skills required		

The absence of a systematic methodology for deciding whether a biomedical data set requires FAIRification led the FAIRplus consortium to develop a series of identification, evaluation, and prioritisation procedures. Selection is achieved by assessing several dimensions: (i) current status of the data and resources needed for FAIR implementation; (ii) ensuring that FAIRification goals meet intended use and reuse scenarios; and (iii) any scientific and socioeconomic impacts arising from FAIRification. The tools for assessment and selection of suitable data sets are complemented by resources supporting the practical implementation of the FAIRification process itself, which will be reported in a future study. We acknowledge that the approach taken has been mainly retrospective; this makes the FAIR process complicated or even impossible, for example because of data loss or poor data management, highlighting the need for prospective FAIR planning and efficient data management.

## Methodology for data prioritisation for FAIRification

**Fig. 1:**
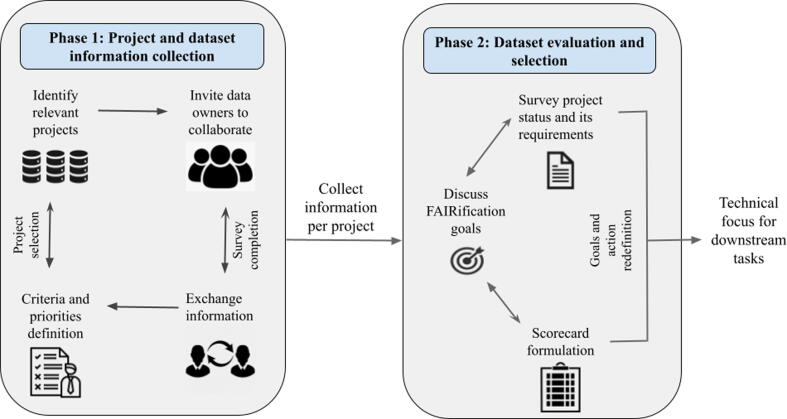
Overview of the workflow for selection of relevant FAIRification projects.

To prioritise biomedical research data for FAIRification, robust identification and evaluation procedures were developed based on practical experience gained from working across 15 IMI projects covering discovery to clinical stages (Figure 1). Given that investment in FAIRification must also support its own strategic goals, FAIRplus prioritised collaboration opportunities with IMI projects that would lead to a high scientific or societal impact. This approach also enables representation of the challenges faced by the wider community and, hence, these learnings could be codified in the FAIR cookbook and generally applied.

**Phase 1: Project and data set information collection**

The information collection phase was carried out in two main steps. First, the identification of relevant projects was conducted using high-level public data describing their scientific activities. This initial identification was followed by a detailed analysis of individual project datasets in collaboration with the data owners.

The starting point for the identification of projects with data resources suitable for FAIRification was text mining from websites and catalogues provided by the IMI (https://www.imi.europa.eu/projects-results/project-factsheets). Basic parameters were extracted, including project start and end dates, participating academic and European Federation of Pharmaceutical Industries and Associations (EFPIA) partners, the contact information of individuals, and a summary of the scientific focus and objectives of each consortium. Pilot-stage evaluations showed that having access to the project-associated personnel with knowledge of the data and its scientific context was a key aspect in deciding FAIRification success. Therefore, projects were grouped into ‘ongoing’, ‘close-to-completion’, and ‘completed’. Active projects with engaged counterparts were later identified. FAIRplus sought out projects working on ‘antimicrobial resistance’, ‘neurodegenerative disease’, ‘healthy aging’, and ‘chronic disease’, to align with its own strategic objective to maximise societal impact. Ensuring strategic alignment with organisational objectives is a general requirement of data set prioritisation efforts. We made use of a Python code for this step of the pipeline. This code is available on GitHub at https://github.com/Fraunhofer-ITMP/IMI-Project-Prioritization/tree/v1.0.

Following initial prioritisation based on strategic alignment with FAIRplus objectives and ability to engage data owners, individual IMI project data resources were analysed to establish the technical, methodological, and ethical features of the data for the establishment of a complete picture of the project, starting from the requirements of the project to the product to be disseminated. Information collected included potential impacts of project and outputs, such as the innovation level (methodological), the comprehensiveness (size), the data availability and accessibility, as well as possible legal aspects associated with the data access, transfer, and/or manipulation. A Data Transfer Agreement (DTA) was developed for use in projects processing sensitive data to provide a legal framework for the processing and reuse of data as part of a FAIRification effort. Additional factors quantified included project maturity, whether data had been made public via other mechanisms, such as publications, or any Intellectual Property (IP) requirements that necessitated that data access would be restricted. The overall FAIR awareness of the consortia was established, which was important in terms of understanding the long-term uptake of the FAIRification measures by the team. One crucial factor was to ensure that collection and processing of all data was carried out in accordance with ethical guidelines and the terms of any informed consent requirements.

**Phase 2: Data set evaluation and selection**

Using the information from the data set analysis, projects were scored across three criteria: societal, scientific, and methodological/technical with a range of 1–4, where 1 was defined as low impact and 4 as high. Societal value was rated according to the research focus of the project and alignment with the FAIRplus strategic priorities. For scientific value, the uniqueness and novelty of the process, the potential synergies across the community, domain coverage, data quality, and the in-house availability of a project or data champions were assessed. Methodological maturity scoring was based on the access model and DMP alongside technical factors, including availability of machine-readable data, presence and status of metadata, the extent of ontology application, data models, workflow documentation, and so on. In situations in which sensitive data were not accessible, their substitution with synthetic data was evaluated. Following the scoring and ranking process by the FAIRplus consortium, 15 of more than 120 IMI projects were prioritised for further engagement and collaborations were initiated around data resource FAIRification. The findings from the ongoing FAIRification work on these projects, including the use of tools for capability assessment and recipes for elevating the FAIRness of specific data resources, are outside the scope of this paper. The template of the scorecard used by FAIRplus during this process can be found on Zenodo.^41^ The impact of decision-making based on the resources is described in detail in the FAIRplus cookbook recipe at https://w3id.org/faircookbook/FCB055.

## Concluding remarks

Previously, studies have outlined the necessity for the adoption of FAIR principles in the pharmaceutical industry, emphasising the high-level financial, technical, and associated cultural challenges.13., 14., 15. Nevertheless, on a practical level, when biomedical-focussed organisations implement FAIR, data owners and managers must determine which resources should be prioritised for FAIRification, as well as the costs versus benefits balance for individuals, institutions, or research communities. Here, we have identified factors that influence the selection and prioritisation of data for FAIR implementation and describe how these can be assessed and used to prioritise projects and data resources, using the experiences of the IMI FAIRplus projects as an example. Given the limited types of exemplar data set that have undergone the FAIRplus FAIRification process, it is envisaged that other types of data set would likely pose a different set of challenges.

Legal and ethical issues have been identified as important factors and, therefore, should be assessed before the FAIRification process because they might require compliance with certain requirements that, if not met or inadequately met, might result in an obstacle for FAIRification. Organisational aspects, including access to domain experts with the technical understanding of the data sets, are also important. Although the methods described mainly come from the translational medicine and drug discovery areas, the challenges, factors, and methodology can be generalised and adapted to other research and development areas; thus, we recommend that researchers and organisations consider the factors suggested here before implementing FAIRification of their data sets.
